# Specificity of Primers and Probes for Molecular Diagnosis of *Leishmania* (*Leishmania*) *chagasi* in Dogs and Wild Animals

**DOI:** 10.3390/pathogens14101065

**Published:** 2025-10-21

**Authors:** Giovanna Zandonadi Haber, Leila Dias da Costa, Erick Bruno Monteiro Costa, Railton Farias Araújo, Tainá Negreiros de Souza, Luciana do Rêgo Lima Queiroz, Bruno Tardelli Nunes Diniz, Edivaldo Costa Sousa Junior, Lívia Carício Martis, Patrícia Karla Santos Ramos, Fernando Tobias Silveira

**Affiliations:** 1 Parasitology Department, Evandro Chagas Institute (Health and Environment Surveillance Secretariat, Ministry of Health), Ananindeua 67030-000, Pará, Brazil; 2 Arbovirus Department, Evandro Chagas Institute (Health and Environment Surveillance Secretariat, Ministry of Health), Ananindeua 67030-000, Pará, Brazil; 3 Tropical Medicine Nucleus, Federal University of Pará, Belém 66055-240, Pará, Brazil

**Keywords:** visceral leishmaniasis, qPCR, primers, TaqMan probe, molecular diagnosis, dogs, wild animals

## Abstract

Molecular tools, especially real-time polymerase chain reaction (qPCR), are relevant tools for laboratory diagnosis due to their sensitivity, specificity, rapid results, and ability to quantify parasite load. This study evaluated the specificity of the LEISH-1/LEISH-2 primer pair with the TaqMan MGB probe in serum samples previously classified by indirect Enzyme-Linked Immunosorbent Assay (ELISA) (30 positive dogs, 30 negative dogs; 9 positive wild animals and 16 negative wild animals) using in silico analyses (Primer-BLAST, Multiple Alignment using Fast Fourier Transform-MAFFT^®^, Geneious, RNAfold, and SnapGene) and Real-Time Polymerase Chain Reaction (qPCR) experimentation. Unexpected amplification occurred in all negative samples, revealing critical specificity failures mainly associated with the probe. In silico analyses confirmed these findings, indicating structural incompatibilities and low selectivity of the sequences. To address this limitation, a new set of oligonucleotides, named GIO, was designed. Computational analyses showed superior performance of GIO, with greater structural stability, absence of unfavorable secondary structures, and improved specificity. Although experimental validation is still required, the results suggest that GIO has strong potential for use in more robust and reliable diagnostic protocols for visceral leishmaniasis across different epidemiological contexts.

## 1. Introduction

American visceral leishmaniasis (AVL) is a serious, non-contagious zoonosis caused by the obligate intracellular protozoan *Leishmania* (*Leishmania*) *chagasi* Lainson & Shaw, 2005 (=*L. chagasi* Cunha & Chagas, 1937), which preferentially parasitizes cells of the mononuclear phagocytic system [1,2,3]. Transmission occurs predominantly through the bite of infected female *phlebotominae Lutzomyia longipalpis* [4]. Although initially restricted to rural areas, American visceral leishmaniasis (AVL) has progressively spread to urban environments in Brazil, driven by environmental and demographic transformations of anthropogenic origin, consolidating itself as a growing challenge for national public health [5,6,7]. Control measures face limitations due to the wide distribution of the vector and the high prevalence of infected domestic dogs, the main urban reservoir. Additionally, socioeconomic inequalities, limited access to timely diagnosis, and the complexity of treatment strategies contribute to the persistence and expansion of the disease in endemic regions [8].

Globally, the World Health Organization (WHO) recognizes AVL as endemic in 80 countries, with Brazil, India, Kenya and Sudan concentrating approximately 68% of reported cases. In the context of the Americas, Brazil is responsible for around 94% of new registrations, as well as having the highest fatality rate attributed to the disease [9]. In the state of Pará, the situation is particularly worrying between 2020 and 2024, 573 new cases were reported, especially in the municipalities of Barcarena and the southeastern region of the state (Marabá, Parauapebas, Canaã dos Carajás and Curionópolis), which together accounted for 175 confirmed cases in the same period [10]. Despite its epidemiological relevance, the diagnosis of AVL still faces significant obstacles. Serological techniques, such as the indirect enzyme-linked immunosorbent assay (indirect ELISA), although widely used in routine diagnosis and surveillance programs, can present limitations in terms of sensitivity and specificity, especially in reservoir hosts and asymptomatic individuals [11]. In this context, molecular methods, especially real-time polymerase chain reaction (qPCR), have emerged as highly relevant tools for laboratory diagnosis. This technique combines high sensitivity and specificity with rapid results and allows the parasite load to be quantified. These characteristics reinforce its potential not only for individual diagnostic confirmation, but also as an epidemiological surveillance strategy, thus consolidating it as a promising alternative to the limitations of conventional methods [12].

However, the accuracy of qPCR assays is directly related to the appropriate design of the primers and probes used. Flaws in the design of these oligonucleotides can compromise the specificity of the reaction, leading to the generation of false-positive results and affecting the reliability of the data obtained [13]. In this way, performing in silico analyses—such as multiple sequence alignments, secondary structure prediction and specificity assessment using bioinformatics tools—is a critical step in the development of robust molecular assays, allowing for the early identification of inconsistencies and the rational optimization of reagents prior to bench application [14,15].

In this context, this study evaluated the specificity of the LEISH-1/LEISH-2 primer set, associated with the TaqMan^®^ MGB Probe [3], for the detection of *Leishmania* (L.) *chagasi* in serum samples from domestic dogs and wild animals previously screened by indirect ELISA. In silico analyses and qPCR assays revealed critical specificity flaws, especially related to the probe, which showed amplification in negative samples. In view of these results, a new set of oligonucleotides was developed, called GIO, which demonstrated superior performance in predictive analyses, showing greater structural stability, absence of unfavorable secondary structures and improved specificity. These characteristics reinforce the potential of the GIO set for application in future qPCR assays aimed at the molecular diagnosis of AVL in different epidemiological contexts.

Therefore, given the limitations in specificity observed in previously described tests and the need to improve diagnostic tools applicable to different epidemiological realities, this study developed and validated a qPCR protocol for the molecular diagnosis of *Leishmania* (L.) *chagasi* in serum samples from domestic dogs and wild animals from endemic and non-endemic areas in the state of Pará.

## 2. Materials and Methods

### 2.1. Study Area and Biological Sample

A total of 85 serum samples were analyzed using the qPCR technique, from different locations in the southeastern region of the state of Pará, Brazil (Graph of the study region). All the samples had previously been screened by Indirect Immunofluorescence Assay (IFAT) [16] and indirect ELISA, making up three experimental groups:

- Group 1 (*n* = 30): domestic dogs with positive serology, from the municipality of Barcarena, collected between 2011 and 2012 as part of the study “Prevalence of canine infection caused by *Leishmania* (L.) *chagasi* determined by PCR in lymph node aspirates”;
- Group 2 (*n* = 30): domestic dogs with negative serology, also from Barcarena and from the same study.
- Group 3 (*n* = 25): samples of wild animals with varying serology, collected between 2020 and 2024 in the municipalities of Parauapebas, Curionópolis, Canaã dos Carajás and Marabá. These municipalities are part of the area of influence of major mining projects in the Carajás Complex (S11D, Serra Leste, Azul and Salobo) and were included in the study “Assessment of environmental and social changes and their influence on the nosological picture”.

For the molecular tests, the *Leishmania* (L.) *chagasi* reference strain MCAO/BR/2010/M27840, isolated from Barcarena (Pará), was used as a positive control. The no template control (NTC), containing only ultrapure water, was used as a negative control. Only samples with (1) defined origin; (2) documented clinical and/or serological history; (3) adequate volume and preserved physical-chemical integrity were included in the analysis.

Samples with (1) insufficient volume; (2) signs of degradation or contamination; (3) no information on origin or clinical/serological history were excluded. All samples were properly stored at −20 °C until molecular processing.

### 2.2. Indirect Enzyme-Linked Immunosorbent Assay (Indirect ELISA)

The detection of specific immunoglobulin G (IgG) antibodies was carried out using indirect ELISA, using polystyrene microplates with 96 wells (flat-bottom) previously sensitized with soluble *Leishmania* (L.) *chagasi* amastigote antigen at a concentration of 10 µg/mL, determined by the Bradford (1976) colorimetric method [17]. The plates were incubated with the antigen diluted in carbonate-bicarbonate buffer (pH 9.6) and kept at 4 °C for 18 h. After sensitization, the wells were blocked with 10% bovine serum albumin (BSA) solution in phosphate-buffered saline (PBS) with pH 7.4 for 1 h at 37 °C, to prevent non-specific binding.

The serum samples were diluted 1:400 in PBS containing 0.05% Tween 20 (PBS-T) and added to the wells in duplicate and incubated at 37 °C for 1 h. After sequential washes with PBS-T, the specific anti-canine or anti-rodent IgG conjugate was added, both linked to alkaline phosphatase (1:2000 dilution), followed by a new incubation under the same conditions. For marsupials, it was not possible to use specific conjugate due to commercial unavailability.

The enzymatic reaction was revealed by adding the chromogenic substrate para-nitrophenylphosphate (pNPP) in carbonate-bicarbonate buffer (pH 9.8). The reaction was carried out for 30 min at room temperature, in a dark environment, being interrupted by the addition of a stop solution when necessary. The optical density (OD) was read on a microplate spectrophotometer at 405 nanometer (nm).

The results were considered positive when the mean OD of the samples was equal to or greater than three standard deviations above the mean values of the negative controls [18]. Serum samples from dogs from non-endemic areas (negative controls) and dogs with confirmed infection by parasitological, serological (IFAT and indirect ELISA), molecular (qPCR) methods and a clinical picture compatible with visceral leishmaniasis (positive controls) were used as controls.

### 2.3. Culture of Leishmania sp. Promastigostes

The isolate of *Leishmania* (L.) *chagasi* used as a positive control in the qPCR assays corresponds to strain MCAO/BR/2010/M27840, from the municipality of Barcarena, Pará, Brazil. This isolate was obtained from the cryobank of the Leishmaniasis Laboratory “Prof. Dr. Ralph Lainson” of the Evandro Chagas Institute (IEC), linked to the Parasitology Section of the Health and Environmental Surveillance Secretariat of the Ministry of Health, Brazil. The promastigote forms were initially cultivated in Neal, Novy and Nicolle (NNN) biphasic medium, incubated at 27 °C until exponential growth began. They were then transferred to Roswell Park Memorial Institute (RPMI) 1640 liquid medium (without phenol red), adjusted to pH 7.2 and supplemented with 10% inactivated fetal bovine serum and 1% antibiotic solution (penicillin/streptomycin), to obtain parasites in the stationary growth phase.

After three consecutive passages, the promastigote forms were washed for three cycles with PBS (14 mM NaCl, 2.5 mM NaH_2_PO_4_-H_2_O, 7.4 mM Na_2_HPO_4_; pH 7.2) and centrifuged at 750 g for 5 min. The resulting pellet was stored at −20 °C until further use [19].

### 2.4. Deoxyribonucleic Acid (DNA) Extraction/Quantification/Quality from Serum

DNA was extracted from positive cultures and serum samples from domestic dogs and wild animals, using the commercial kit ReliaPrep gDNA Tissue Miniprep System (Promega, Madison, WI, USA) [20]. Initially, the samples were subjected to cell lysis with proteinase K and then transferred to a column containing a silica membrane. During centrifugation, the DNA adhered to the membrane and was then purified through successive washing steps. At the end of the process, the DNA was eluted in 150 µL of elution buffer. The samples were quantified using a Qubit 2.0 Fluorometer (Invitrogen, Carlsbad, CA, USA) using a DNA concentration of between 5 and 10 ng/µL for molecular biology assays.

### 2.5. Evaluation of Serum Samples from Domestic Dogs and Wild Animals by qPCR

To evaluate the 85 serum samples from domestic dogs and wild animals, sensitivity and specificity tests were carried out to verify the ability of the primers and probe to detect *Leishmania* (L.) *chagasi* DNA.

The qPCR reaction controls included NTC, composed only of ultrapure water, and a positive control, represented by a standard reference strain of *Leishmania* (L.) *chagasi* (MCAO/BR/2010/M27840), at a dilution of 1:1,000,000. All controls were processed in triplicate to monitor the specificity of the reaction.

The qPCR reactions were carried out with 500 nM of the LEISH-1 (5′-AACTTTCTGGTCCTCCGGGTAG-3′) and LEISH-2 (5′-ACCCCCAGTTTCCCGCC-3′) primers, as well as 200 nM of the TaqMan^®^ MGB probe (FAM-5′-AAAAATGGGTGCAGAAAT-3′-NFQ-MGB; Applied Biosystems, USA). The Cq (quantification cycle) values obtained for each dilution were plotted against the logarithm of the relative DNA concentration to construct the standard curve.

The set of primers and probe (LEISH-1/LEISH-2/TaqMan^®^ MGB probe) were not newly designed for this study. Instead, they were retrieved from previous publications [3] where they had been validated for *Leishmania* detection. And they evaluated in this study were selected based on its prior use in molecular diagnosis of *Leishmania* sp. and on reported sensitivity for the detection of kinetoplast DNA sequences. This choice was motivated by its potential applicability to serum samples, which often contain low parasitic loads, requiring highly sensitive molecular tools. By testing this primer–probe set, we aimed to establish a reliable reference point for comparison with additional primer designs. This approach allowed us to both validate existing tools under new experimental conditions and to develop an optimized primer–probe set (GIO) tailored for improved sensitivity.

The reaction mix was prepared with: nuclease-free water and 4 μL of DNA, totaling a final volume of 20 μL per reaction; containing 0.5 μM of each primer; 10 μL of GoTaq^®^ Probe qPCR Master Mix (2×); and 2 μL of CXR Reference Dye per mL of master mix. The thermal cycling conditions were initially denaturation at 95 °C for 2 min, followed by 40 cycles of denaturation at 95 °C for 15 s and annealing/extension at 60 °C for 1 min. The reactions were conducted on the AriaMx Real-Time PCR System (Agilent Technologies, Santa Clara, CA, USA) [3,21].

The Cq (Cycle Quantification) values obtained in duplicates were plotted on a graph to make it easier to visualize and interpret the results. To do this, a boxplot graph was drawn up, in which all the samples were organized according to their respective groups. The figure was constructed using the R programming language and RStudio software version 2025.09.0, ensuring greater clarity and objectivity in the comparative analysis between the groups evaluated.

### 2.6. In Silico Analysis of Primer and Probe Molecular Structures

The in silico analysis was performed using the GenBank sequence MT598578.1, representative of *Leishmania infantum/chagasi*.

To evaluate the specificity of the primers and probes used in the qPCR assay, an in silico analysis of the oligonucleotide sequences (forward, reverse, and probe) was conducted.

Initially, Primer-BLAST (NCBI)^®^ was employed. This tool integrates the basic local alignment search tool (BLAST) algorithm with primer design parameters to identify potential matches with genomic sequences available in public databases, as well as possible cross-reactivity with related species, such as other *trypanosomiases* [14,15].

Subsequently, the sequences were formatted in FASTA and aligned using MAFFT^®^ (Multiple Alignment using Fast Fourier Transform) version 7.526. Multiple sequence alignment was performed with kDNA minicircle regions from *Leishmania* (L.) *chagasi* strains (GenBank ID: MT598578.1), using the -auto parameter, which automatically selects the most suitable alignment algorithm. This step enabled the assessment of whether the oligonucleotides were anchored in highly conserved and specific regions of *Leishmania* (L.) *chagasi*, or if they targeted regions shared with other species, which would compromise diagnostic specificity [22].

Additionally, Geneious^®^ software version 2025.2 was used to analyze the physicochemical properties of the oligonucleotides, including melting temperature (Tm), GC content (%), thermal stability, sequence length, and the potential for dimer formation and hairpin-like secondary structures. These structures were further visualized using the RNAfold Web Server^®^, as such features are critical for ensuring both the efficiency and specificity of qPCR reactions.

Finally, SnapGene^®^ software version 7.6.1 (GSL Biotech LLC, Chicago, IL, USA) was employed to simulate PCR reactions and agarose gel electrophoresis, to verify the expected size of the amplicons generated by both the previously published Leish primers and the newly designed GIO set. Both primer sets were developed based on conserved regions of the *Leishmania* (L.) *chagasi* kDNA minicircle [23,24].

### 2.7. Data Analysis and Statistics

Statistical analyses were performed using BioEstat^®^ software, version 5.0 [25], with a 95% confidence level (*p* < 0.05) considered significant. For the 85 samples evaluated—both positive and negative for AVL—Fisher’s Exact Test was applied to compare the frequency of qPCR-positive and—negative results between domestic and wild animals.

The main diagnostic performance parameters were calculated: sensitivity, specificity, positive predictive value (PPV), accuracy, F1-score, and Cohen’s Kappa coefficient. The latter was used to assess the level of agreement between qPCR and indirect ELISA results, with interpretation based on the following criteria:

- Kappa < 0: disagreement
- 0 to 0.20: slight agreement
- 0.21 to 0.40: fair agreement
- 0.41 to 0.60: moderate agreement
- 0.61 to 0.80: substantial agreement
- 0.81 to 1.00: almost perfect agreement

These statistical analyses were essential to validate the reliability and diagnostic accuracy of the qPCR assay, supporting its applicability for the detection of Leishmania DNA in different host species.

## 3. Results

### 3.1. Evaluation of Serum Samples from Domestic Dogs and Wild Animals by Indirect ELISA and qPCR

A total of 85 serum samples were analyzed using an indirect IgG ELISA, including 60 samples from domestic dogs and 25 from wild animals. In Group 01 (*n* = 30), composed of dogs from an endemic area for visceral leishmaniasis and previously testing positive by IFAT-IgG, all samples showed reactivity in the indirect ELISA, resulting in 100% positivity. In contrast, Group 02 (*n* = 30), consisting of dogs from a non-endemic area and negative by IFAT-IgG, showed no reactivity in the assay. Group 03, composed of 25 wild animals from endemic regions, showed indirect ELISA positivity in 36% (9/25) and negativity in 64% (16/25), as presented in Table 1.

**Figure 1:**
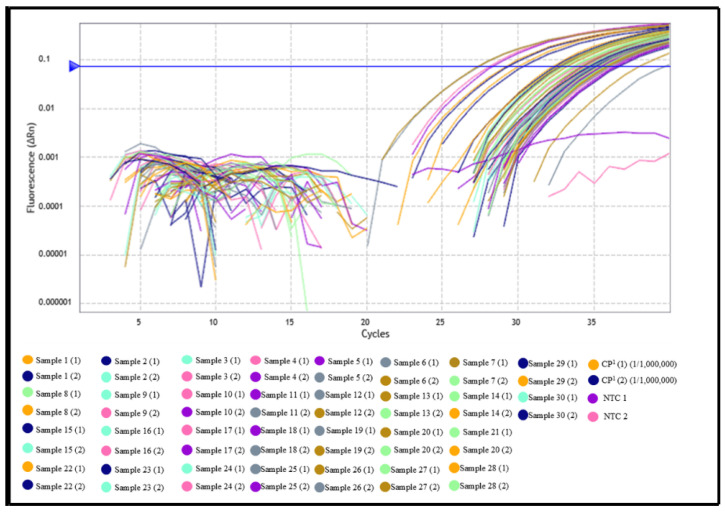
Clinical performance evaluation of the qPCR assay for detection of *Leishmania* (L.) *chagasi* in serum samples. Amplification curves from the clinical sensitivity test using positive control DNA (MCAO/BR/2010/M27840—Barcarena, Pará) and sera from ELISA-confirmed positive dogs, showing amplification with Cq values ranging from 22 to 34. NTC: no-template control.

**Figure 2:**
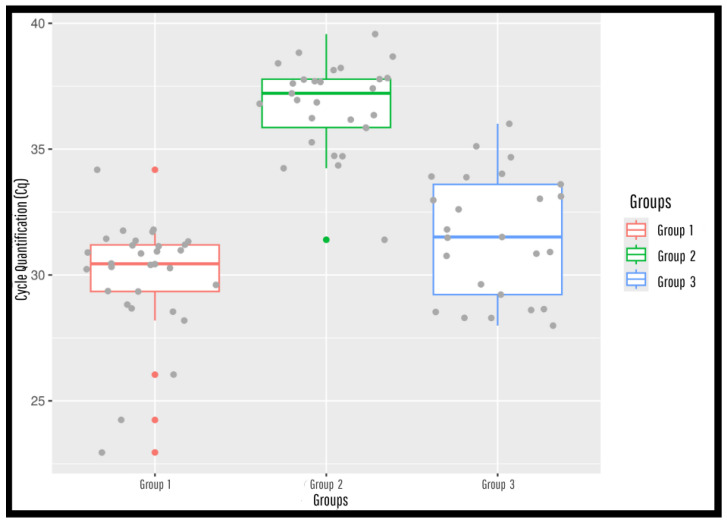
Box plot graphic of serological and molecular evaluation of serum samples from domestic dogs and wild animals for the detection of *Leishmania* (*Leishmania*) *chagasi* using the R programming language and the software RStudio version 2025.09.0. Samples were collected from both endemic and non-endemic areas in Pará State-Brazil—Group 01 are 30 positive dogs from Barcarena; group 02, are 30 negative dogs from Belém Metropolitan Region; group 03 are 25 wild animals from Terra Alta, Marabá, Canaã dos Carajás, Curianópolis and Parauapebas-, and tested using ELISA-IgG and qPCR assays. Results include qualitative qPCR status (positive/negative) and quantitative amplification parameters (Cq values and ΔRn).

Serum samples from Group 01 showed real-time polymerase chain reaction amplification in 100% of cases, with Cq values ranging from 22.95 to 34.18, resulting in a molecular test sensitivity of 100% (Figure 1 and Figure 2, Supplementary Table S1). The average Cq value of the positive control was 24.94, which is consistent with varying parasitic loads and indicates the absence of false positives.

**Figure 3:**
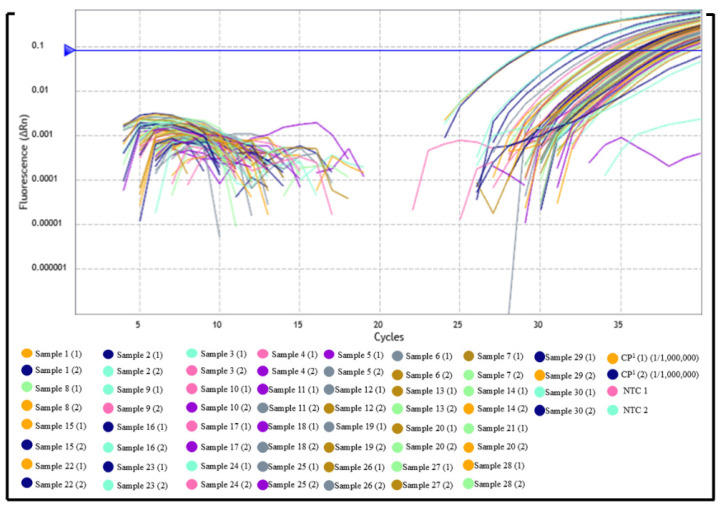
Clinical performance evaluation of the qPCR assay for detection of *Leishmania* (L.) *chagasi* in serum samples. Amplification curves from the clinical specificity test using sera from ELISA-negative dogs, revealing unexpected amplification in most samples, suggesting possible false-negative results or misclassification by indirect ELISA.

On the other hand, among the samples in Group 02, previously classified as negative by indirect ELISA, 29 samples showed amplification by qPCR (Cq ranging from 31.00 to 39.00), while only one sample did not amplify (Figure 2 and Figure 3, Supplementary Table S1).

**Figure 4:**
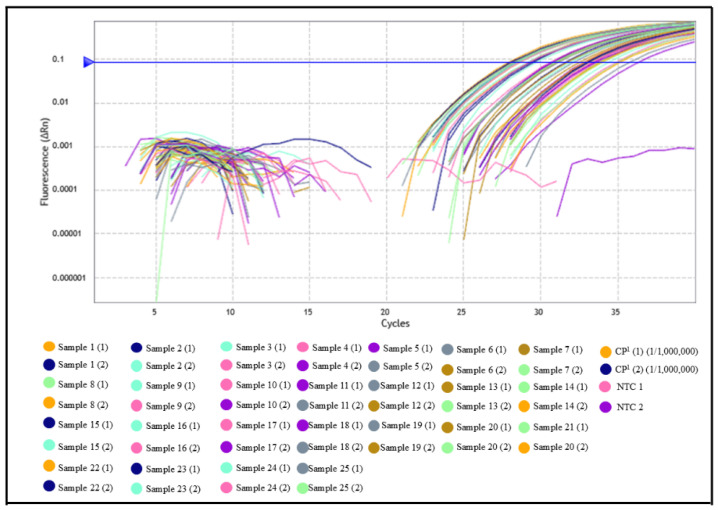
Clinical performance evaluation of the qPCR assay for detection of *Leishmania* (L.) *chagasi* in serum samples. qPCR amplification profiles from serum samples of wild animals (foxes, rodents, and marsupials), including both ELISA-positive and ELISA-negative specimens, all exhibiting amplification with Cq values between 27 and 36, highlighting the assay’s broad detection capacity in sylvatic reservoirs. Pará State, Brazil, 2025.

This finding resulted in a clinical specificity of only 2.17%, suggesting possible detection of residual parasitic DNA, subclinical infection, or limitations of the serological assay. In Group 03, composed of wild animals, all samples showed amplification by qPCR, with Cq values ranging from 27.99 to 36.01, despite only 9 samples testing positive by indirect ELISA (Figure 2 and Figure 4 and Supplementary Table S1). This discrepancy between the molecular and serological methods contributed to the low agreement observed between the two assays.

### 3.2. In Silico Analysis of Primer and Probe Sequences

The LEISH-1/LEISH-2 primers showed 100% identity and coverage with the reference sequence of the *Leishmania* (L.) *donovani/infantum* kDNA minicircle (GenBank: MT598578.1). The E-value for the LEISH-1 primer was 0.023, which is considered statistically significant, indicating strong binding to conserved and specific regions of the *donovani complex*. In contrast, the LEISH-2 primer exhibited a high E-value (88), suggesting low binding affinity compared to LEISH-1 (Table A1). In Silico BLAST analysis revealed significant alignments exclusively with *Leishmania infantum/chagasi* (GenBank accession MT598578.1), with no hits detected for other *Trypanosomatidae* species. A summary of the BLAST output is provided in Supplementary File S1.

**Figure 5:**
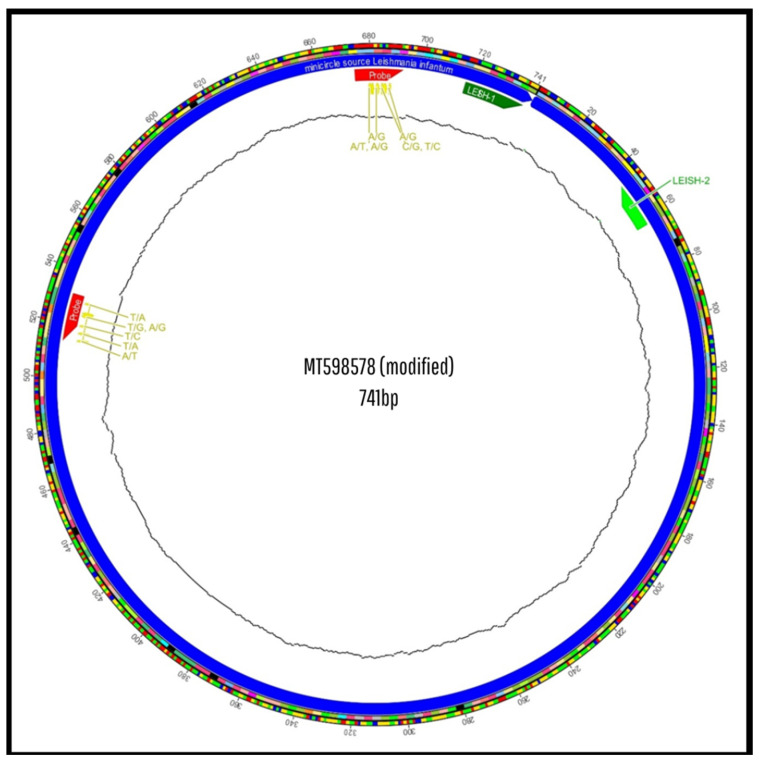
In Silico alignment of the *Leishmania* (L.) *chagasi* kDNA minicircle sequence. Multiple sequence alignment showing the positions of previously used primers (LEISH-1, LEISH-2) and the TaqMan^®^ MGB probe, highlighting mismatches and incompatibilities that may contribute to nonspecific amplification. Alignments were generated using MAFFT^®^ software version 7.526 and based on the reference sequence GenBank ID: MT598578.1. Pará State, Brazil, 2025.

Although the TaqMan^®^ MGB probe was designed from the same reference sequence, it did not exhibit any identity or coverage hits and was classified as having no specific target (Table A1). Multiple sequence alignment performed using MAFFT^®^ (Figure 5) revealed several SNPs and misalignment of the probe, which was positioned outside the region defined by the primers. Notably, polymorphisms were detected at positions 603 (A/T), 604 (A/G), 605 (A/G), 607 (C/G), 608 (T/C) in the probe binding region, likely to explain the occurrence of non-specific amplification in ELISA-negative samples and highlighting potential compromises in both detection efficiency and specificity.

The analysis of the physicochemical properties of the oligonucleotides (Table A2) revealed a significant thermal imbalance between the primers: LEISH-1 had a melting temperature (Tm) of 67.5 °C, while LEISH-2 had a Tm of 57.5 °C and a low GC content (33.3%), resulting in a 10 °C difference. The probe (17 Base Pairs, 70.6% GC) presented a Tm of 69.9 °C, which is considered excessively high for its length, violating qPCR design guidelines that recommend similar Tm values between primers and a probe Tm 5 to 10 °C higher than that of the primers [4,26].

**Figure 6:**
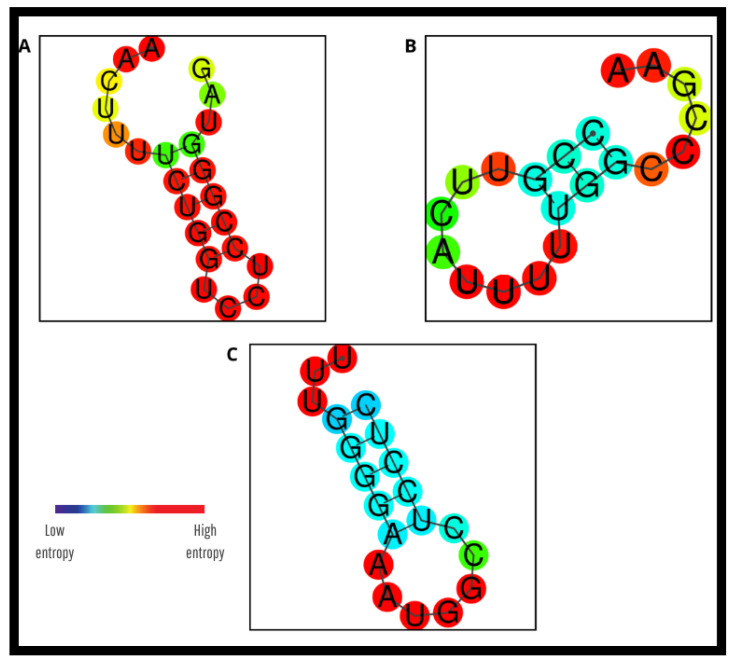
Predicted secondary structures of oligonucleotides used in qPCR for detection of *Leishmania* (L.) *chagasi*, obtained through in silico hairpin formation analysis. (**A**) Structure of primer LEISH-1; (**B**) Structure of primer GIO-06F; (**C**) Structure of probe GIO-96P. The color gradient (blue to red) indicates base-pairing probability or positional entropy, where more inclined toward blue represents stable/consistent regions and more inclined toward red reflects unstable or variable sites. Predictions were performed using Geneious^®^ software version 2025.2 and the RNAfold Web Server online tool. Pará State, Brazil, 2025.

**Figure 7:**
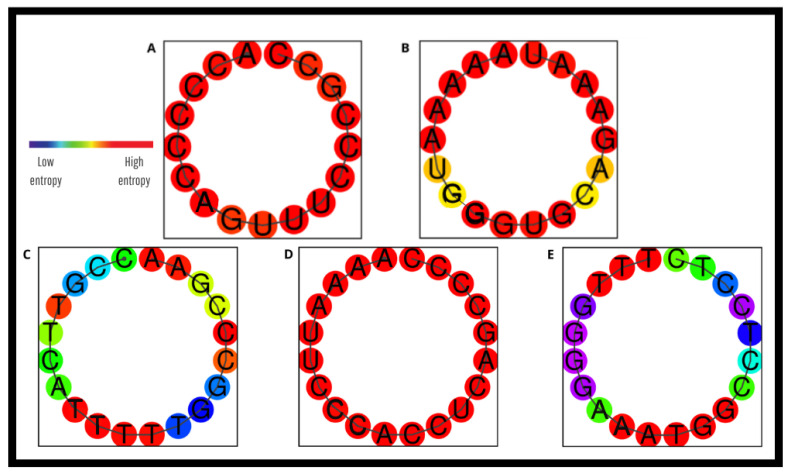
Predicted secondary structure of the oligonucleotides used in qPCR for detection of *Leishmania* (L.) *chagasi,* obtained by in silico analysis of primer dimer formation. (**A**) Structure of LEISH-2 primer; (**B**) Structure of TaqMan^®^ MGB Probe; (**C**) Structure of GIO-06F primer; (**D**) Structure of GIO-155R primer; (**E**) Structure of GIO-96P probe. The color gradient (blue to red) indicates base-pairing probability or positional entropy, where more inclined toward blue represents stable/consistent regions and more inclined toward red reflects unstable or variable sites. Predictions were made using Geneious^®^ software version 2025.2 and the online RNAfold Web Server tool. Pará, Brazil, 2025.

Regarding secondary structure formation, the predicted structures were generated using RNAfold^®^, where the color gradient from blue to red represents either base-pairing probability or positional entropy. In the case of base-pairing probability, blue indicates highly stable and reliable pairings, while red corresponds to uncertain or unpaired regions. When visualized by positional entropy, blue reflects low entropy, meaning the structure is highly consistent, whereas red indicates high entropy and structural variability Thus, blue regions denote stable and predictable conformations, while red highlights flexible or unstable sites. A hairpin was identified in the LEISH-1 primer, with a Tm of 41.1 °C (Figure 6A). Additionally, primer-dimer formations involving LEISH-2 and the probe were observed (Figure 7A,B), which may negatively affect the reaction by compromising assay efficiency This structures was considered irrelevant due to its melting temperature being well below that of the primer itself and the color red reinforces this, since the redder it is, the higher the entropy and structural variability [5,6,7].

**Figure 8:**
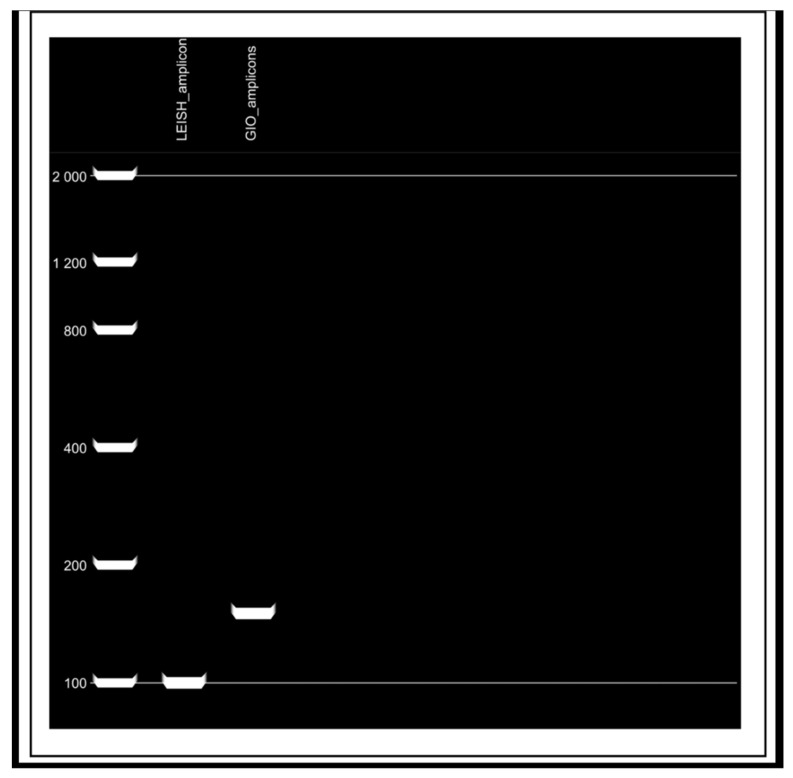
Simulated agarose gel electrophoresis for estimation of amplicon sizes obtained with LEISH and GIO primer pairs. A molecular weight marker (100–2000 bp) was used as a reference for fragment size estimation. (LEISH_amplicon) Migration profile of fragments amplified with LEISH primers (LEISH-1 and LEISH-2), showing bands corresponding to the expected size of 100 bp, according to the molecular weight marker. (GIO_amplicon) Migration profile of fragments amplified with GIO primers (GIO-06F and GIO-155R), showing bands corresponding to the expected size of 150 bp, according to the molecular weight marker. Analyses were performed using SnapGene^®^ software version 7.6.1. Pará State, Brazil, 2025.

PCR and agarose gel electrophoresis simulations performed using SnapGene^®^ (Figure 8) indicated that the LEISH-1/LEISH-2 primers amplify a fragment of approximately 100 Base Pairs (bp). This amplicon size falls within the optimal range for qPCR assays (70–200 bp), which favors high amplification efficiency and reliable detection, particularly in samples with degraded DNA or low parasitic load [4,26].

### 3.3. In Silico Evaluation of the New Primers and Probe

**Figure 9:**
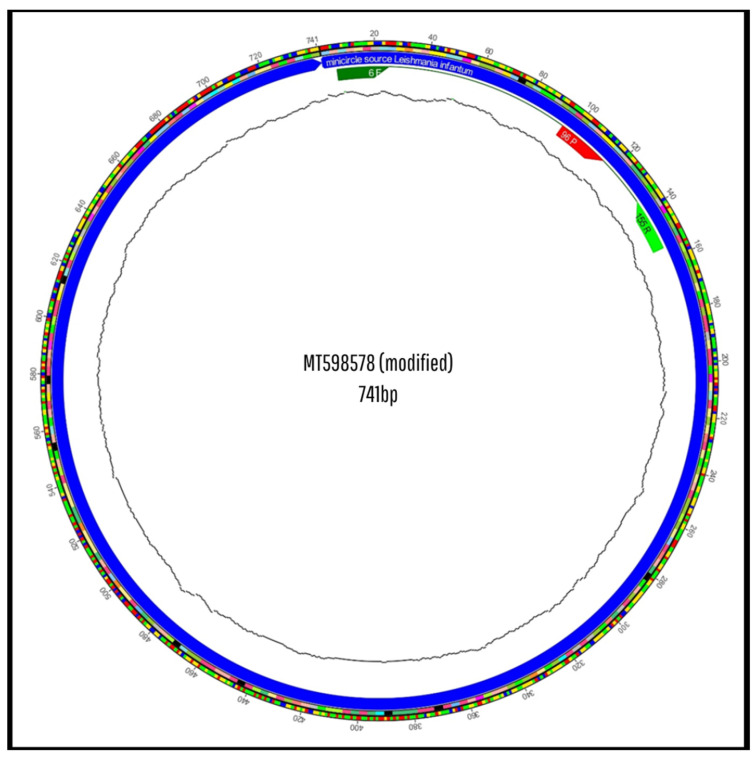
In Silico alignment of the *Leishmania* (L.) *chagasi* kDNA minicircle sequence. Alignment with newly designed primers (GIO-06F and GIO-155R) and probe (GIO-96P), demonstrating precise positioning in conserved target regions, optimized spacing, and absence of polymorphisms at hybridization sites. Alignments were generated using MAFFT^®^ software version 7.526 and based on the reference sequence GenBank ID: MT598578.1. Pará State, Brazil, 2025.

To overcome the limitations observed with the previous oligonucleotides, new molecular markers were developed: GIO-06F (forward primer), GIO-155R (reverse primer), and GIO-96P (probe). All consist of 20 bp with melting temperatures (Tm) ranging from 59.7 °C to 60.3 °C, GC content between 50% and 55%, hairpin formation melting temperatures between 28.8 °C and 33.6 °C, and primer-dimer formation potentials occurring at temperatures well below their Tm (approximately 4 °C) (Figure 6B,C, Figure 7C–E and Figure 9; Table A2).

Unlike the original set, these new oligonucleotides exhibit greater thermal compatibility and a reduced risk of artifacts. Additionally, an improved predicted performance of the GIO-96P probe, especially for samples with low parasitic loads, such as those derived from wild animals [5,6,7]. Nonetheless, the use of TaqMan^®^-MGB probes is recommended to increase the Tm of short probes, ensuring hybridization prior to DNA polymerase extension [4].

Specificity analysis using Primer-BLAST (NCBI) demonstrated that all oligonucleotides have 100% identity and coverage with the reference sequence of the *Leishmania* (L.) *donovani/infantum* kDNA minicircle (GenBank accession number: MT598578.1), with highly significant E-values and scores (Table A3), confirming their suitability for molecular diagnostic applications.

In the genomic alignment conducted with MAFFT^®^ (Figure 9), it was observed that the GIO-06F primer starts at position 6, and the GIO-155R primer is located between bases 155 and 136, resulting in the amplification of a fragment approximately 150 bp in length. The GIO-96P probe, positioned between the two primers (positions 96–115), is correctly placed to hybridize prior to extension, according to established criteria for TaqMan^®^ probe-based assays [4,5,6,7].

PCR reaction simulation performed using SnapGene^®^ software version 7.6.1. (Figure 8) showed that the new primer set (GIO) generates an amplicon of approximately 150 bp, which falls within the recommended range for qPCR assays (70–200 bp). Although the amplicon size is slightly larger than that of the previous set, this fragment offers a potential advantage in terms of specificity. Prior evaluations of its physicochemical and structural properties reinforce the suitability for accurate detection of *Leishmania* (L.) *chagasi* [4,26].

### 3.4. Statistical Analysis

Furthermore, statistical tests were conducted, including the evaluation of sensitivity, specificity, and other diagnostic performance parameters. Comparing ELISA and qPCR using Fisher’s Exact Test on the 85 serum samples, we observed *p* = 1.00, this qualitatively confirms that all ELISA-negative samples also tested positive in qPCR, making the association non-significant. The overall accuracy of the qPCR compared to indirect ELISA was 47.06%, while the positive predictive value (PPV) reached 46.42%. The F1-score, representing the harmonic mean of precision and sensitivity, was 63.41%, reflecting the contrast between the high sensitivity (100%) and the low precision of the test.

Cohen’s Kappa coefficient was 0.019, indicating minimal agreement between the evaluated methods. These findings suggest that, although qPCR was effective in detecting all true positives, it exhibited a high rate of false positives, particularly among seronegative dogs and wild animals, which negatively impacted the test’s specificity and precision (Table 1).

## 4. Discussion

American visceral leishmaniasis (AVL) is a neglected zoonosis of significant public health importance, responsible for high morbidity and mortality in humans and widely distributed across tropical and subtropical regions. In Brazil, the Northern region—particularly the state of Pará—exhibits high endemicity, necessitating continuous improvements in surveillance and control strategies. In this context, early identification of infection in human, canine, and wild reservoirs is critical to interrupting transmission chains and reducing disease burden.

Rather than being a single linear approach, our study integrated multiple strategies for primer–probe evaluation. We first assessed a widely used primer–probe set from the literature, followed by the design and validation of a new set (GIO). This in silico analysis provided preliminary insights into the performance of existing tools versus newly proposed alternatives, suggesting possible strengths and limitations that warrant further experimental validation for molecular detection of *Leishmania* in animal serum samples.

Serological methods such as indirect ELISA and IFAT are widely employed due to their simplicity and relatively low cost. However, these assays are limited by reduced sensitivity and specificity, especially during early infection stages or in animals exhibiting low immune responses [27,28]. The qPCR has emerged as a promising alternative, offering higher sensitivity, precise quantification, and direct detection of parasitic DNA even in complex matrices like serum [27,28]. Nevertheless, the diagnostic performance of qPCR is heavily dependent on the quality of oligonucleotides used, necessitating rigorous validation of primers and probes for specificity, amplification efficiency, and thermochemical compatibility [1,29].

In this study, the LEISH oligonucleotide set yielded 100% sensitivity in ELISA-positive dogs but only 2.17% clinical specificity, as nearly all ELISA-negative samples were also amplified by qPCR. This lack of concordance between assays was also evident in wild animal samples, where 100% amplification occurred by qPCR despite only 36% positivity in ELISA. These findings indicate that the low agreement observed is unlikely to be due solely to differences in immune response but rather suggest cross-reactivity or limited probe specificity, consistent with our in silico predictions. Such discrepancies reinforce the need for designing and validating more specific primers and probes, such as the newly proposed GIO set.

In this study, we critically evaluated the performance of the traditional oligonucleotide set described by Francino et al. [3]—LEISH-1/LEISH-2 primers and TaqMan^®^ MGB probe—for detecting *Leishmania* (L.) *chagasi* in serum samples from dogs and wild animals previously characterized by indirect ELISA. The results demonstrated 100% sensitivity in ELISA-positive dogs but only 2.17% clinical specificity, as nearly all ELISA-negative samples were also amplified by qPCR, resulting in Fisher’s Exact Test with *p* = 1.00, overall accuracy of 47.06% and a Cohen’s Kappa coefficient of 0.019, which indicates minimal agreement between methods. A similar lack of concordance was observed in wild animal samples, where qPCR detected 100% amplification despite only 36% positivity in ELISA.

These findings indicate that the lack of statistical association between ELISA and qPCR results cannot be explained solely by immune response variability but are more likely due to cross-reactivity or limited probe specificity, consistent with our in silico predictions [11,12]. The unexpected amplification observed in ELISA-negative samples further supports the hypothesis of probe misalignment and non-specific binding [14,24,30]. Together, these results highlight the importance of integrating in silico analyses with empirical validation, as they confirm the critical limitations of the traditional LEISH set and emphasize the need for designing and validating more specific primers and probes, such as the newly proposed GIO set.

It is important to note that the primer–probe set initially selected (LEISH-1/LEISH-2/TaqMan^®^ MGB probe) was chosen due to its extensive use and validation in previous studies of visceral leishmaniasis [3,21]. For this reason, no in silico evaluation was conducted prior to our experimental testing. Interestingly, our findings revealed that, despite its broad use, this set did not amplify serum samples efficiently, underscoring the importance of re-evaluating even widely applied molecular tools when adapting them to different sample types or diagnostic settings. This unexpected outcome highlights the relevance of our study, as it demonstrates the necessity of combining both experimental and comparative approaches when selecting primers for diagnostic purposes [6,31].

Although the primers showed satisfactory genomic compatibility in silico, the TaqMan^®^ MGB probe exhibited critical structural flaws: lack of full identity and coverage with the target sequence, presence of multiple polymorphisms (Figure 5), thermal imbalance (disproportionately high Tm), and improper positioning relative to primers. Such inadequacies likely compromise assay specificity by promoting nonspecific hybridization, resulting in background fluorescence, amplification of off-target sequences, and consequent diagnostic inaccuracy [30,31]. Similar concerns regarding probe design impacting qPCR specificity have been reported in other parasitological studies, emphasizing the need for careful oligonucleotide design and validation [27,32].

These findings underscore the necessity of robust in silico validation steps prior to qPCR oligonucleotide design, including comprehensive genomic alignments across diverse *Leishmania donovani* complex strains and species, polymorphism analysis, secondary structure prediction, and thermochemical evaluation. Adherence to MIQE guidelines (Minimum Information for Publication of Quantitative Real-Time PCR Experiments) [1] is crucial to ensure reliability and reproducibility in molecular assays. Previous studies corroborate that qPCR assays following MIQE recommendations demonstrate significantly improved diagnostic accuracy and inter-laboratory consistency [33,34].

In response to these limitations, we developed a novel oligonucleotide set, designated GIO (comprising primers GIO-06F and GIO-155R and probe GIO-96P), designed from the reference kDNA minicircle sequence of *Leishmania* (L.) *infantum/chagasi* (GenBank: MT598578.1). In Silico analyses confirmed that the GIO set has 100% identity and coverage with the target sequence, strategic positioning within conserved regions, absence of significant secondary structures, and balanced Tm values among primers and probe. Additionally, the 150 bp amplicon falls within the optimal qPCR size range (70–200 bp), enhancing amplification efficiency in samples with low DNA concentrations, such as serum from wild animals [3,5,6,7].

It should be noted that BLAST analysis, made for both sets of primers and probes, retrieved hits only with *L. infantum/chagasi*, and no alignments were detected for other *trypanosomatid* species, this indicates a degree of sequence-level specificity, especially considering the LEISH set [35].

Based on these data, it can be inferred that the poor performance of the traditional set is not solely attributable to experimental factors but primarily to structural deficiencies in the oligonucleotides, especially the probe. Therefore, replacement with rationally validated alternative sets like GIO represents a promising advancement toward developing more reliable molecular protocols suitable for routine diagnosis and epidemiological surveillance of AVL.

While experimental validation of the GIO set is necessary to confirm its diagnostic performance, the predictive results presented here are an important step toward standardizing more analytically robust molecular targets. Such efforts are essential not only to improve diagnostic accuracy, but also to strengthen AVL control and prevention measures in endemic regions.

From a public health perspective, improving molecular diagnostic tools like qPCR is vital for timely detection and management of AVL reservoirs, which directly impact disease transmission dynamics. Accurate identification of infected animals, especially asymptomatic or early-stage cases, can guide targeted interventions, optimize resource allocation, and support One Health approaches that integrate human, animal, and environmental health considerations [36]. Moreover, molecular assays with enhanced specificity reduce the risk of unnecessary treatments and minimize the psychological and economic burden on affected communities.

Future studies should focus on experimental validation of the GIO primer/probe set in diverse field samples, assessment of its performance across different biological matrices, and comparison with other molecular and serological assays. Integration of molecular diagnostics into routine surveillance programs will require capacity building, standardization of protocols, and cost-effectiveness analyses to ensure sustainable implementation in endemic areas.

## 5. Conclusions

This study provided a comprehensive and critical evaluation of the LEISH-1/LEISH-2 primer set combined with the TaqMan^®^ MGB Probe, originally developed by Francino et al. (2006) [3], exposing significant and previously underappreciated limitations in assay specificity. Detailed in silico analyses uncovered critical structural deficiencies—including marked thermal mismatches, suboptimal probe positioning, and incomplete sequence identity with the target kDNA minicircle—that collectively explain the elevated false-positive rates and the poor concordance observed when compared to indirect ELISA results. These findings align with concerns raised in other molecular diagnostic studies emphasizing the pivotal role of primer and probe design in ensuring assay accuracy and reliability.

In response to these limitations, we designed a novel oligonucleotide set, termed GIO (GIO-06F, GIO-155R, and GIO-96P), employing rigorous bioinformatic strategies to optimize sequence specificity, thermodynamic properties, and secondary structure stability. The GIO set demonstrated exemplary in silico performance, including perfect genomic alignment with the *Leishmania* (L.) *chagasi* reference sequence, well-balanced melting temperatures, and absence of deleterious secondary structures, all of which are fundamental parameters that, based on in silico evidence, support their potential suitability for quantitative PCR assays. Importantly, the amplicon size of approximately 150 bp falls within the optimal range for qPCR, particularly benefiting detection in challenging samples such as serum from wild animals with low parasitic loads.

By addressing critical weaknesses of the existing primer–probe system and offering a rationally optimized alternative, this study advances the molecular toolkit available for visceral leishmaniasis diagnosis. The GIO set’s predicted improvements in specificity and amplification efficiency may substantially enhance diagnostic accuracy and reduce false-positive outcomes, pending experimental validation, which are crucial for both clinical decision-making and epidemiological surveillance.

The robust bioinformatic validation presented here lays a strong foundation for future experimental verification and field validation. Once empirically confirmed, the GIO oligonucleotides have the potential to become a cornerstone of molecular diagnostics in endemic regions, contributing to more reliable case detection, improved disease monitoring, and better-informed control strategies against AVL. Overall, these findings represent a significant step forward in standardizing and strengthening qPCR methodologies for Leishmania detection, addressing a critical gap in the current diagnostic landscape.

## Figures and Tables

**Table 1:** Serological evaluation of domestic dog and wild animal sera by ELISA-IgG, stratified by endemic and non-endemic areas and RIFI status, for the detection of *Leishmania* (L.) *chagasi* infection in Pará, Brazil.

Samples	Samples (N)	Region/IFAT *	Positive (%)	Negative (%)
Domestic Dogs	60	Endemic (IFAT * IgG ** +)	30/30 (100%)	0/30 (0%)
		Non-Endemic (IFAT * IgG ** −)	0/30 (0%)	30/30 (100%)
Wild Animals	25	Endemic	9/25 (36%)	16/25 (64%)

* IFAT: Indirect Immunofluorescence Assay; ** IgG: Immunoglobulin G.

## Data Availability

The raw data supporting the conclusions of this article will be made available by the authors on request.

## Supplementary Materials

The following supporting information can be downloaded at: https://www.mdpi.com/article/10.3390/pathogens14101065/s1, Table S1: Serological and molecular evaluation of serum samples from domestic dogs and wild animals for the detection of Leishmania (Leishmania) chagasi. Samples were collected from both endemic and non-endemic areas in Pará State, Brazil, and tested using ELISA-IgG and qPCR assays. Results include qualitative qPCR status (positive/negative) and quantitative amplification parameters (Cq values and ΔRn); File S1: Output from the NCBI Primer-BLAST tool showing the in silico BLAST results for the LEISH primers and the TaqMan^®^ MGB probe and GIO set (primers and probe). The file includes alignment details, specificity analysis, and confirmation of exclusive matches with Leishmania infantum/chagasi sequences, with no cross-reactivity detected for other Trypanosomatidae species. The article of Francino et al., 2006. Advantages of real-time PCR assay for diagnosis and monitoring of canine leishmaniasis; Can be downloaded at: https://pubmed.ncbi.nlm.nih.gov/16473467 (accessed on 24 July 2025).

## Abbreviations

The following abbreviations are used in this manuscript:

AVL	American Visceral Leishmaniasis
ELISA	Indirect Enzyme-Linked Immunosorbent Assay
MAFFT^®^	Multiple Alignment using Fast Fourier Transform
WHO	World Health Organization
qPCR	Real-Time Polymerase Chain Reaction
NTC	No Template Control
IFAT	Indirect Immunofluorescence Assay
pNPP	Chromogenic Substrate Para-Nitrophenylphosphate
IEC	Evandro Chagas Institute
NNN	Neal, Novy and Nicolle
Tm	Melting Temperature
bp	Base Pairs
PPV	Positive Predictive Value
IgG	Immunoglobulin G
BSA	Bovine Serum Albumin
PBS	Phosphate-Buffered Saline
PBS-T	Phosphate-Buffered Saline Tween
OD	Optical Density
nm	Nanometer
RPMI	Roswell Park Memorial Institute
DNA	Deoxyribonucleic acid
USA	United State of America
Cq	Cycle Quantification
BLAST	Basic Local Alignment Search Tool

## Appendix A

**Table A1:** Comparative analysis of the sequence alignment of primers LEISH-1 and LEISH-2 and the TaqMan^®^ MGB probe used in qPCR for the detection of *Leishmania* (L.) *chagasi*. The analysis was performed using the NCBI Primer-BLAST tool and includes identity (%), coverage, alignment score, and E-value. GenBank accession number: MT598578.1. Pará State, Brazil, 2025.

Parameter	Primer Forward (LEISH-1)	Primer Reverse (LEISH-2)	Probe (TaqMan^®^-MGB Probe)
**Query**	LEISH-1	LEISH-2	Probe TaqMan^®^
**Sequence ID (Subject)**	MT598578.1	MT598578.1	MT598578.1
**Organism**	*L. donovani*/*infantum*	*L. donovani*/*infantum*	*L. donovani*/*infantum*
**% Identity**	100%	100%	-
**Query cover**	100%	100%	-
**Score (Bit score)**	46.1	34.2	-
**E-value**	0.023	88	-
**Alignment** **(Start-End)**	Start-717/End-739	Star: 63/End-47	-
**Expected target** **(Yes/No)**	Yes	Yes	No

**Table A2:** Comparative analysis of oligonucleotides used in qPCR assays for the detection of *Leishmania* (*Leishmania*) *chagasi*, including GC content (%), predicted hairpin structures, sequence length (bp), and melting temperature (Tm, °C). The evaluation includes newly designed primers and probe (GIO-06F, GIO-155R, GIO-96P) and previously tested oligonucleotides (LEISH-1, LEISH-2, and TaqMan^®^ MGB probe). Nucleotide sequences are confidential and not disclosed. In Silico analysis was conducted using Geneious^®^ software version 2025.2. Pará State, Brazil, 2025.

Name	%GC	Type	Hairpin (°C)	Sequence Size	Melting Temperature (Tm°C)
GIO-06F	50.0%	Primer	28.8	20	59.7
GIO-96P	55.0%	Probe	33.6	20	60.0
GIO-155R	55.0%	Primer	-	20	60.3
Leish-1	52.2%	Primer	41.1	23	67.5
TaqMan^®^-MGB Probe	70.6%	Probe	-	17	69.9
Leish-2	33.3%	Primer	-	18	57.5

**Table A3:** Comparative analysis of the sequence alignment of primers GIO-06F and GIO-155R and the probe GIO-96P used in qPCR for the detection of *Leishmania* (L.) *chagasi*. The analysis was performed using the NCBI Primer-BLAST tool and includes identity (%), coverage, alignment score, and E-value. GenBank accession number: MT598578.1. Pará State, Brazil, 2025.

Parameter	Primer Forward (GIO-06F)	Primer Reverse (GIO-155R)	TaqMan^®^-MGB Probe (GIO-96P)
**Query**	GIO-06F	GIO-155R	GIO-96P
**Sequence ID (Subject)**	MT598578.1	MT598578.1	MT598578.1
**Organism**	*L. donovani*/*infantum*	*L. donovani*/*infantum*	*L. donovani*/ *infantum*
**% Identity**	100%	100%	100%
**Query cover**	100%	100%	100%
**Score (Bit score)**	40.1	40.1	40.1
**E-value**	1.4	1.4	1.4
**Alignment** **(Start-End)**	Start-06/End-25	Start-155/End-136	Start-96/End-115
**Expected target** **(Yes/No)**	Yes	Yes	Yes
